# Unstable intertrochanteric fractures are associated with a greater hemoglobin drop during the perioperative period: a retrospective case control study

**DOI:** 10.1186/s12891-020-03208-2

**Published:** 2020-04-15

**Authors:** Po-Hsun Lin, Jui-Teng Chien, Jung-Pin Hung, Chih-Kai Hong, Tzung-Yi Tsai, Chang-Chen Yang

**Affiliations:** 1 Department of Orthopedics, Dalin Tzu Chi Hospital, Buddhist Tzu Chi Medical Foundation, No. 2, Min-Sheng Road, Dalin Town, Chia-Yi, Taiwan; 2 Institute of Medical Sciences, Tzu Chi University, Hualien, Taiwan; 3 School of Medicine, Tzu Chi University, Hualien, Taiwan; 4 Department of Orthopedic Surgery, National Cheng Kung University Hospital, College of Medicine, National Cheng Kung University, Tainan, Taiwan; 5 Department of Medical Research, Dalin Tzu Chi Hospital, Buddhist Tzu Chi Medical Foundation, No. 2, Min-Sheng Road, Dalin Town, Chia-Yi, Taiwan; 6 Department of Nursing, Tzu Chi University of Science and Technology, Hualien, Taiwan

**Keywords:** Intertrochanteric fractures, Unstable fractures, Hemoglobin drop, Perioperative complications, AO classification

## Abstract

**Background:**

With an increase in the elderly population, the occurrence of hip fractures, femoral neck fractures, and intertrochanteric fractures (ITFs) is also increasing. It is important to establish effective perioperative methods that would help reduce the morbidity and mortality rates associated with ITFs. The purpose of this study was to determine the effects of ITFs according to the AO classification for perioperative hemoglobin drop.

**Methods:**

Seventy-six patients with ITFs classified as AO 31-A1 or A2 and fixated with intramedullary nails participated in this retrospective cohort study. Medical records of these patients were retrospectively reviewed from September 2016 to August 2018. The perioperative hemoglobin drop was chosen as the main outcome measure and calculated as the difference between pre- and postoperative hemoglobin levels. Multivariate linear regression analysis was performed and included the following variables: AO classification (A1.1-A2.1 [stable] vs. A2.2-A2.3 [unstable]), time interval between injury and surgery, age, body mass index, and the use of anticoagulants.

**Results:**

Among the 76 patients who met the inclusion criteria, a significantly higher hemoglobin drop was observed in the AO 31 A2.2-A2.3 (unstable) group than in the AO 31 A1.1-A2.1 (stable) group (*p* = 0.04). The multivariate analysis also showed a greater hemoglobin drop in the unstable group (*p* < 0.05).

**Conclusions:**

Patients with unstable ITFs exhibited a greater hemoglobin drop and a hidden blood loss was suspected around the fracture site. We believe that this should be taken into consideration when presurgical blood transfusion is being planned for patients with unstable ITFs, to reduce associated postoperative complications, especially in patients with severe anemia or high risk of mortality.

## Background

Due to advances in healthcare, the elderly population is increasing worldwide. The incidence rate of hip fractures is also expected to increase to over 6 million fractures per year by the year 2050 [1]. This likely increase in hip fractures includes femoral neck fractures and intertrochanteric fractures (ITFs) [2].

The mortality and morbidity in patients with ITFs are significantly higher than in those with femoral neck fractures, and this is most likely due to the older age and the corresponding fragile physical conditions of patients who experience ITFs [1]. ITFs are known to cause more complications (such as pneumonia and anemia) and postoperative mortality than femoral neck fractures [3]. Thus, reducing mortality and morbidity rates after ITF surgery, with an emphasis on improving perioperative care, is important for increasing the overall survival [4].

Hip fractures can result in obvious blood loss; more specifically, patients with ITFs may have an underestimated blood loss, which increases postsurgical morbidity [5–7]. However, initial hematocrit (Hct) or hemoglobin (Hb) screening may seem normal on admission of these patients to the emergency department [5–7]. The accurate estimation of hidden blood loss may predict the Hb drop after fracture fixation, which could potentially improve the overall survival of patients with ITF.

Risk factors that may have an impact on hidden blood loss, such as age, presence of an unstable fracture, intramedullary fixation, and general anesthesia, have been reported [5–7].

Since an Hb drop during the perioperative period may increase the risk of mortality and morbidity in patients undergoing surgery for ITFs, it is critical to determine the important risk factors for preventing the development of postoperative anemia. However, few studies have assessed the association between the AO classification and Hb drop in ITFs. Therefore, in this study, we focused on the impact of fracture type, according to the AO classification and hidden blood loss, which was evaluated by measuring the perioperative Hb drop. We hypothesized that unstable fractures according to the AO classification would show a greater perioperative Hb drop.

## Methods

### Patient eligibility

All patients treated with open reduction with internal fixation from September 2016 to August 2018 were retrospectively reviewed. A total of 231 patients who experienced proximal femoral fractures were reviewed at the Department of Orthopedics of the Buddhist Dalin Tzuchi Hospital, Taiwan. Ethical approval was obtained from the Institutional Review Board and the Ethics Committee of the Buddhist Dalin Tzuchi Hospital, Taiwan Hospital (IRB number: B10801009). The requirement for patients’ informed consent was waived due to the retrospective study design. Inclusion criteria included the presence of an ITF classified as AO 31-A1 or A2 and a fixation with intramedullary nail. Patients were excluded if they had a femoral neck, subtrochanteric, or proximal femoral shaft fracture, fixation with sliding hip screw, a blood transfusion between injury and postoperative Hb follow-up, less than 60 years of age, or a pathological or AO 31-A3 fracture.

ITFs were defined according to the AO classification [8]. They were categorized into two groups as follows: [1] the stable group, which included AO 31-A1.1 through AO 31-A2.1, and [2] the unstable group, which included AO 31-A2.2 through AO 31-A3.3. In this study, only the stable group(AO 31-A1.1 through AO 31-A2.1) and the unstable group (AO 31-A2.2 through AO 31-A2.3) were discussed.

### Data collection

Three independent observers who were blinded to the surgical treatment defined the fracture type on the preoperative X-rays, using the modified AO classification. Patient data, including preoperative and postoperative Hb levels, age, sex, weight, height, body mass index (BMI), time interval between injury and surgery, duration of operation, surgeon, occurrence of blood transfusions, bone mineral density (BMD), American Society of Anesthesiologists (ASA) classification, underlying diseases, and medication history, were retrospectively recorded from the medical chart. The Hb drop was calculated based on the difference in the Hb level between the first emergency room record and the record from the morning after surgery. When patients with ITF were admitted to our ward for surgery, they were kept on a nothing by mouth regimen. Although vital signs, consciousness, respiratory rate, electrocardiograph, blood pressure, or urinary output were not presented in detail in this study, all patients were treated to have these parameters maintained within the normal range based on the Vital Signs Directed Therapy Protocol and adequate urine output (> 0.5 cc/kg/hour). Moreover, lower limb traction was not applied, and no medications (e.g., tranexamic acid) were administered before surgery.

### Statistical analyses

All comparisons between the stable and unstable groups were made using independent samples t-tests. Multiple linear regression analysis was used to evaluate the risk factors affecting Hb drop. Distribution normality and independence of variables were tested using the Shapiro-Wilk and Durbin-Watson tests, respectively. All statistical analyses were performed using IBM SPSS 20 (IBM, Chicago, IL, USA). A *p*-value < 0.05 was considered statistically significant.

## Results

**Fig. 1:**
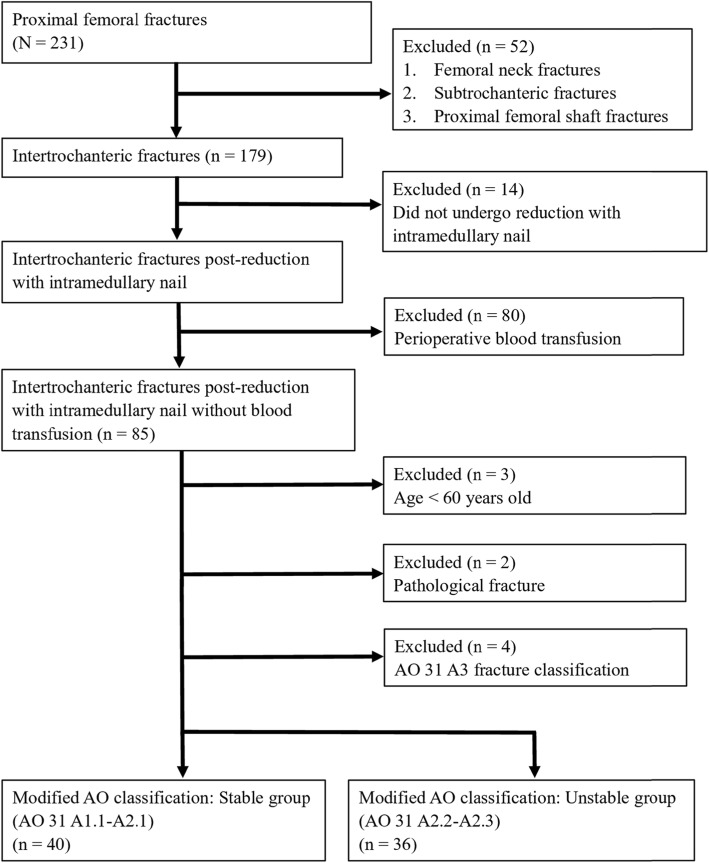
Flow chart of study design

A total of 231 patients with proximal femoral fractures were identified; 155 of these patients were excluded because they had a different fracture site (52 patients), fixation with other systems (14 patients), pathological fractures (2 patients), were undergoing blood transfusion (80 patients), were less than 60 years of age (3 patients) and had different fracture classification (4 patients, AO 31-A3, Fig. 1).

Seventy-six patients were included in this study. The mean age of these patients was 80.56 years (range, 64–96 years) and 27 patients were male. According to the AO classification, 40 patients were in the stable group (A1.1 - A2.1) and 36 patients were in the unstable group (A2.2 - A2.3). All fractures were fixed with intramedullary nails. The characteristics of patients with stable and unstable ITFs are displayed in Table 1. The mean pre- and postoperative Hb levels for the stable and unstable groups were 12.1 g/dl and 9.0 g/dl, respectively. The average time interval between injury and surgery was 18 h and the mean duration of operation was 78.5 min. The mean BMI was 23.14 kg/m^2^. There were no significant differences between the two groups with regard to sex, age, time interval between injury and surgery, operative time, BMD, ASA classification, BMI, and the number of patients taking anticoagulants. However, the Hb drop was significantly higher in the unstable group (*p* = 0.04).

**Table 1:** Patient demographic data

	Stable group^a^	Unstable group^a^	Whole cohort^a^	*p*-value
AO classification	AO 31 A1.1 - A2.1	AO 31 A2.2 - A2.3	–	
Number of patients	40	36	76	
Sex			–	0.70
Male	15	12		
Female	25	24		
Age (years);			Mean age: 80.56 years	0.91
60 ≤Age≤ 70	3	3		
70 <Age≤ 80	16	12		
80 <Age≤ 90	21	21		
Hb (g/dl)				
Preoperative Hb	11.82	12.51	12.1	0.08
Postoperative Hb	8.93	9.12	9.0	0.62
Hb drop	2.89	3.39	3.1	0.04*
Time between injury and surgery				0.77
< 24 h	37	32	18	
> 24 h	3	4		
Duration of operation;				0.33
Time < 60 min	14	10		
60 min ≤Time< 120 min	22	22	78.5	
Time ≥ 120 min	4	4		
T-score			–	0.15
No record	6	9		
T-score ≥ -1	5	1		
-2.5 ≤T − score< -1	11	18		
T-score < -2.5	18	8		
ASA classification			–	0.41
2	2	5		
3	38	30		
4	0	1		
BMI (kg/m^2^);			23.14	0.58
BMI < 18.5	5	5		
18.5 ≤BMI< 24	22	17		
BMI ≥ 24	13	14		
Anticoagulant use			–	0.94
Yes	1	1		
No	39	35		

Abbreviations: *ASA* American Society of Anesthesiologists, *BMI* Body mass index, *Hb* Hemoglobin

Definitions: Stable group = AO 31 A1.1-A2.1, Unstable group = AO 31 A2.2–2.3, Preoperative Hb = first hemoglobin data recorded in emergency room

Postoperative Hb = hemoglobin data recorded in the morning after surgery,

*p < 0.05

^a^Mean values for each parameter are listed

The results of the multiple linear regression analysis are shown in Table 2. The factors influencing Hb drop included fracture classification, time interval between injury and surgery, age, BMI, and use of anticoagulants. The unstable group had a significantly higher Hb drop after adjusting for other factors (β = 0.51; p = 0.04). The time interval between injury and surgery revealed no significant difference in Hb drop (*p* = 0.10). Age, BMI, and the use of anticoagulants also showed no significant differences between groups (*p* = 0.10, 0.36, and 0.42, respectively). We also confirmed that our regression model was normally distributed (*p* = 0.42), independent (Durbin-Watson test = 1.91), and had constant variance. There was no co-linearity between the independent variables.

**Table 2:** Multiple linear regression analysis of hemoglobin drop after adjusting for confounding variables

Multiple linear regression analysis				
	*β*	t	*p* value	VIF
AO classification (stable and unstable)	0.51	2.07	0.04*	1.00
Time between injury and surgery	0.13	1.65	0.10	1.18
Age	0.03	1.64	0.10	1.14
BMI	0.02	0.90	0.36	1.13
Anticoagulant use	−0.67	−0.80	0.42	1.19

Abbreviations: *BMI* Body mass index, *VIF* Variance Inflation Factor

Dependent variable: hemoglobin drop;

Independent variables: AO classification (stable and unstable), time interval between injury and surgery, age, BMI, and anticoagulant use

*p < 0.05

## Discussion

The findings of our study revealed that the unstable ITF group had a significantly higher Hb drop after adjusting for other factors (β = 0.51; *p* = 0.04). There were no significant differences between the two groups with respect to sex, age, time interval between injury and surgery, duration of operation, BMD, ASA classification, BMI, and the number of patients taking anticoagulants. ITFs are one of the most common fractures in the elderly. Patients with ITFs can have blood loss from the fracture itself and can become dehydrated before the fracture is diagnosed and repaired. Therefore, the preoperative Hb level may not reflect the real blood loss and is frequently underestimated [9]. There are some factors that affect the total blood loss after ITF. First, blood loss due to trauma is the most significant reason and likely causes of the greatest Hb drop [9, 10]. Second, the surgical approach also affects the total blood loss; in particular, the blood loss increases when reduction with an intramedullary nail is performed [11]. Intramedullary nail reduction is a common treatment for ITF and it can also cause a greater hidden blood loss than other approaches [11].

Compared to femoral neck fractures, ITFs are extracapsular, which means they are associated with greater blood loss. Blood loss from cancellous bones in ITFs is usually significant [10], and the total blood loss affects the pre- to postoperative Hb drop. Therefore, the Hb drop in ITFs is more obvious than that in femoral neck fractures [10].

There are several risk factors that affect the Hb level when patients sustain ITFs and undergo intramedullary nail fixation [12–14]. A comminuted fracture usually causes more blood loss than a simple fracture [15, 16]. In their case series, Ronga et al. and Torres et al. [15, 16]. reported more blood loss in AO 31-A2 fractures than in AO 31-A1 fractures. In this study, a greater Hb drop was demonstrated in the unstable group, according to the AO classification. The unstable group of ITFs also revealed a greater Hb drop during the perioperative period. The risk factors for greater blood loss and hidden blood loss are age, time interval between injury and surgery, duration of operation, BMI, presence of diabetes mellitus, and the use of anticoagulants [15–20].

In this study, ITFs were defined according to the Sonawane’s criteria [8], which classified them into two groups: AO 31-A1.1 through A2.1 (commonly described as stable) and AO 31-A2.2 through A3.3 (described as unstable). In this study, the AO 31-A3 group was excluded due to the small number of patients and the different trauma biomechanics of patients with this fracture type (fracture line away from the greater and lesser trochanters) compared to AO 31-A1 and A2 fractures. AO 31-A3 fractures are reverse oblique fractures, simple transverse fractures, or shaft and subtrochanteric extensions. Moreover, a study showed that ITFs classified as AO 31-A3 with intramedullary nail reduction have a lower duration of operation and are less likely to require blood transfusion [21]. Therefore, including these fractures in the unstable group might have prevented us from identifying a greater Hb drop.

The unstable group showed a higher Hb drop compared to the stable group (β = 0.51; *p* = 0.04). However, the other analyzed factors showed no significant differences in this study. This might be due to the small number of cases. On the other hand, we excluded all patients who immediately received blood transfusion at the emergency room. Therefore, we could have excluded patients with major blood loss and this could have affected our findings. We also excluded patients < 60 years old because we wanted to focus on the older population, with a higher prevalence of ITF.

Due to the aforementioned risk factors, a greater Hb drop will cause more complications. The Hb drop in the ITF perioperative period is affected by the amount of blood loss due to the fracture and the severity of dehydration [19]. The Hb drop is a significant postoperative complication; previous studies have shown that it may be a predictive factor of mortality rate after ITF [22, 23].

The presence of anemia, either pre- or postoperatively, causes significant effects in older patients after ITF reduction [12, 24–26]. Anemia within the ITF preoperative period and greater perioperative blood loss are poor prognosis factors associated with higher postoperative mortality rate, higher risk of bed-related complications (i.e., pneumonia, urinary tract infections, and deep vein thrombosis), increased length of hospital stay, increased readmission rate, poor physical performance, and poor functional recovery [27, 28]. ITFs have a higher mortality rate than femoral neck fractures, particularly during the first year after discharge [10]. We did not analyze the postoperative follow-up in these patients; therefore, we could not confirm that a higher mortality rate is associated with unstable ITFs. This study could serve as a reference for future studies that will compare the one-year mortality rates between stable and unstable fractures.

The ITF mortality rate has been associated with anemia and surgical delay [29, 30]. The appropriate time for ITF surgery is within 24 to 72 h of the occurrence of trauma [31–33]. Surgery within 48 h of admission after ITF will reduce the length of hospital stay, mortality rate, and perioperative complications [29, 30]. If the length of time between admission and surgery is > 24 h, patients will have lower blood loss. The hematoma formation may produce local pressure at the fracture site to reduce the total blood loss [15]. Postoperative anemia is correlated with an inferior functional recovery and a detrimental effect on mortality. Therefore, aggressive pre-surgical management, such as blood transfusion, may improve the overall outcome in patients who are expected to have more blood loss [34].

Preoperative and trauma-related blood loss are unavoidable; however, if more attention is paid to patients who have a greater bleeding risk and higher complication rate, they can be treated aggressively, for example, with preoperative blood transfusion, intraoperative blood transfusion, and reduced surgery time. This may lead to a better prognosis by reducing postoperative complications, the average length of hospital stay, average medical costs, and one-year mortality rate. However, the preferred transfusion regimen, especially in patients with pre-existing cardiorespiratory diseases, remains controversial [35, 36]. A liberal blood transfusion may result in pro-inflammatory, immunomodulating, and prothrombotic effects [37], which could cause serious systemic complications, such as cardiovascular events. Carow J et al. [38] reported that a liberal blood transfusion causes higher cardiorespiratory complications in patients with trochanteric femoral fractures. We believe that the prediction of Hb drop would be helpful when blood transfusion is being considered in such fragile patients.

The Hb level considered to be a “trigger” for blood transfusion remains controversial, especially in elderly patients [39]. For elderly patients, careful transfusion practices to maintain Hb thresholds within a range of 9–10 g/dl are indicated [39]. Therefore, we could consider aggressive blood transfusion before surgery to reduce postoperative anemia (hemoglobin < 9 g/dl).

Diabetic or other medical statuses may influence Hb values especially in fragile elderly patients. However, this study exclusively focused on the parameters that may have a direct impact on significant hemorrhage, such as fracture type, age, BMI, and the use of anticoagulants; the influences of these medical issues were not evaluated in our study.

Both Hb and Hct are used as evaluation methods for anemia. According to previous studies, Hb level provides a direct measure of the oxygen-carrying capacity while Hct provides an indirect measure [40]. Therefore, Hb level is routinely used for anemia assessment at our institution, and we used this measure in this study.

The small number of cases was the greatest limitation to this study. Patients who underwent blood transfusion and were excluded accounted for about one-third of all cases at the period of initial data collection. The technical performance of the surgeons and their overall experience also affects the outcomes in AO 31-A3 fractures, in terms of reduction in skill and duration of operation. Furthermore, the external validity of this study could only be applied to the older patients (> 60 years old) who had unstable ITFs. Also, due to the small number of cases included in this study, the external validity may be limited by age, AO classification, and number of cases. Future studies should include more cases and a longer follow-up time to assess the mortality rate between a preoperative aggressive blood transfusion group and nonaggressive blood transfusion group.

## Conclusions

The unstable ITF group showed a greater Hb drop and hidden blood loss in the third space than the stable group. Therefore, in ITFs classified as AO 31- A2.2/2.3 in the emergency room, close attention should be paid to the vital signs, underlying diseases, age, BMI, trauma time, and estimated duration of operation of the patients. We believe that this should be taken into consideration when presurgical blood transfusion is being planned for patients with unstable ITFs, to reduce the associated postoperative complications, especially in patients with severe anemia or high mortality risk.

## Data Availability

The datasets used and/or analyzed during the current study available from the corresponding author on reasonable request.

## Abbreviations

ASA: American Society of Anesthesiologists
BMD: Bone mineral density
BMI: Body mass index
Hct: Hematocrit
Hb: Hemoglobin
ITF: Intertrochanteric fracture

## References

[1] Kannus P, Parkkari J, Sievanen H, Heinonen A, Vuori I, Jarvinen M (1996). Epidemiology of hip fractures. Bone.

[2] Karagas MR, Lu-Yao GL, Barrett JA, Beach ML, Baron JA (1996). Heterogeneity of hip fracture: age, race, sex, and geographic patterns of femoral neck and trochanteric fractures among the US elderly. Am J Epidemiol.

[3] Roche JJ, Wenn RT, Sahota O, Moran CG (2005). Effect of comorbidities and postoperative complications on mortality after hip fracture in elderly people: prospective observational cohort study. BMJ..

[4] Kiriakopoulos E, McCormick F, Nwachukwu BU, Erickson BJ, Caravella J (2017). In-hospital mortality risk of intertrochanteric hip fractures: a comprehensive review of the US Medicare database from 2005 to 2010. Musculoskelet Surg.

[5] Li B, Li J, Wang S, Liu L (2018). Clinical analysis of peri-operative hidden blood loss of elderly patients with intertrochanteric fractures treated by unreamed proximal femoral nail anti-rotation. Sci Rep.

[6] Wu JZ, Liu PC, Ge W, Cai M (2016). A prospective study about the preoperative total blood loss in older people with hip fracture. Clin Interv Aging.

[7] Zhang Y, Shen J, Mao Z, Long A, Zhang L, Tang P (2014). Risk factors of hidden blood loss in internal fixation of intertrochanteric fracture. Zhongguo xiu fu chong jian wai ke za zhi = Zhongguo xiufu chongjian waike zazhi = Chin J Reparative Reconstruct Surg.

[8] Sonawane DVJTI (2015). Classifications of Intertrochanteric fractures and their Clinical Importance. Trauma Int.

[9] Kumar D, Mbako AN, Riddick A, Patil S, Williams P (2011). On admission haemoglobin in patients with hip fracture. Injury..

[10] Smith GH, Tsang J, Molyneux SG, White TO (2011). The hidden blood loss after hip fracture. Injury..

[11] Foss NB, Kehlet H (2006). Hidden blood loss after surgery for hip fracture. J Bone Joint Surg.

[12] Bhaskar D, Parker MJ (2011). Haematological indices as surrogate markers of factors affecting mortality after hip fracture. Injury..

[13] Carson JL, Noveck H, Berlin JA, Gould SA (2002). Mortality and morbidity in patients with very low postoperative Hb levels who decline blood transfusion. Transfusion..

[14] Lawrence VA, Hilsenbeck SG, Noveck H, Poses RM, Carson JL (2002). Medical complications and outcomes after hip fracture repair. Arch Intern Med.

[15] Ronga M, Bonzini D, Valoroso M, La Barbera G, Tamini J, Cherubino M (2017). Blood loss in trochanteric fractures: multivariate analysis comparing dynamic hip screw and gamma nail. Injury..

[16] Torres A, Laffosse J, Molinier F, Tricoire J, Chiron P, Puget J (2012). Statistical analysis of the factors that increase perioperative bleeding in trochanteric fractures. Revista espanola de cirugia ortopedica y traumatologia.

[17] Chechik O, Thein R, Fichman G, Haim A, Tov TB, Steinberg EL (2011). The effect of clopidogrel and aspirin on blood loss in hip fracture surgery. Injury..

[18] Reina N, Geiss L, Pailhe R, Maubisson L, Laffosse JM, Chiron P (2014). Traumax screw plate vs. gamma nail. Blood loss in pertrochanteric fractures treated by minimally invasive osteosynthesis. Hip Int J Clin Experim Res Hip Pathology Therapy.

[19] Wang J, Wei J, Wang M (2015). The risk factors of perioperative hemoglobin and hematocrit drop after intramedullary nailing treatment for intertrochanteric fracture patients. J Orthopaed Sci.

[20] Liu Y, Sun Y, Fan L, Hao J (2017). Perioperative factors associated with hidden blood loss in intertrochanteric fracture patients. Musculoskelet Surg.

[21] Sadowski C, Lubbeke A, Saudan M, Riand N, Stern R, Hoffmeyer P (2002). Treatment of reverse oblique and transverse intertrochanteric fractures with use of an intramedullary nail or a 95 degrees screw-plate: a prospective, randomized study. J Bone Joint Surg Am.

[22] Foss NB, Kehlet H (2005). Mortality analysis in hip fracture patients: implications for design of future outcome trials. Br J Anaesth.

[23] Martinez V, Monsaingeon-Lion A, Cherif K, Judet T, Chauvin M, Fletcher D (2007). Transfusion strategy for primary knee and hip arthroplasty: impact of an algorithm to lower transfusion rates and hospital costs. Br J Anaesth.

[24] Carson JL, Duff A, Poses RM, Berlin JA, Spence RK, Trout R (1996). Effect of anaemia and cardiovascular disease on surgical mortality and morbidity. Lancet (London, England).

[25] Hu F, Jiang C, Shen J, Tang P, Wang Y (2012). Preoperative predictors for mortality following hip fracture surgery: a systematic review and meta-analysis. Injury..

[26] Willems JM, de Craen AJ, Nelissen RG, van Luijt PA, Westendorp RG, Blauw GJ (2012). Haemoglobin predicts length of hospital stay after hip fracture surgery in older patients. Maturitas..

[27] Bateman L, Vuppala S, Porada P, Carter W, Baijnath C, Burman K (2012). Medical management in the acute hip fracture patient: a comprehensive review for the internist. Ochsner J.

[28] Foss NB, Kristensen MT, Kehlet H (2008). Anaemia impedes functional mobility after hip fracture surgery. Age Ageing.

[29] Khan SK, Kalra S, Khanna A, Thiruvengada MM, Parker MJ (2009). Timing of surgery for hip fractures: a systematic review of 52 published studies involving 291,413 patients. Injury..

[30] Klestil T, Roder C, Stotter C, Winkler B, Nehrer S, Lutz M (2018). Impact of timing of surgery in elderly hip fracture patients: a systematic review and meta-analysis. Sci Rep.

[31] Aqil A, Hossain F, Sheikh H, Aderinto J, Whitwell G, Kapoor H (2016). Achieving hip fracture surgery within 36 hours: an investigation of risk factors to surgical delay and recommendations for practice. J Orthopaed Traumatol.

[32] Hapuarachchi KS, Ahluwalia RS, Bowditch MG (2014). Neck of femur fractures in the over 90s: a select group of patients who require prompt surgical intervention for optimal results. J Orthopaed Traumatol.

[33] Yonezawa T, Yamazaki K, Atsumi T, Obara S (2009). Influence of the timing of surgery on mortality and activity of hip fracture in elderly patients. J Orthop Sci.

[34] Lawrence VA, Silverstein JH, Cornell JE, Pederson T, Noveck H, Carson JL (2003). Higher Hb level is associated with better early functional recovery after hip fracture repair. Transfusion..

[35] Brunskill SJ, Millette SL, Shokoohi A, Pulford EC, Doree C, Murphy MF, et al. Red blood cell transfusion for people undergoing hip fracture surgery. Cochrane Database Syst Rev. 2015;21(4):CD009699. 10.1002/14651858.CD009699.pub2 PMC11065123 25897628

[36] Chatterjee S, Wetterslev J, Sharma A, Lichstein E, Mukherjee D (2013). Association of blood transfusion with increased mortality in myocardial infarction: a meta-analysis and diversity-adjusted study sequential analysis. JAMA Intern Med.

[37] Twomley KM, Rao SV, Becker RC (2006). Proinflammatory, immunomodulating, and prothrombotic properties of anemia and red blood cell transfusions. J Thromb Thrombolysis.

[38] Carow J, Carow JB, Coburn M, Kim BS, Bucking B, Bliemel C (2017). Mortality and cardiorespiratory complications in trochanteric femoral fractures: a ten year retrospective analysis. Int Orthop.

[39] Goodnough LT, Schrier SL (2014). Evaluation and management of anemia in the elderly. Am J Hematol.

[40] Quinto L, Aponte JJ, Menendez C, Sacarlal J, Aide P, Espasa M (2006). Relationship between haemoglobin and haematocrit in the definition of anaemia. Trop Med Int Health.

